# Fabrication of ZnO Nanoparticle-Decorated Nanofiber Mat with High Uniformity Protected by Constructing Tri-Layer Structure

**DOI:** 10.3390/polym12091859

**Published:** 2020-08-19

**Authors:** Duy-Nam Phan, Hyeong Yeol Choi, Seong-Geun Oh, Myungwoong Kim, Hoik Lee

**Affiliations:** 1 School of Textile-Leather and Fashion, Hanoi University of Science and Technology, 1 Dai Co Viet Road, Hanoi 100803, Vietnam; nam.phanduy@hust.edu.vn; 2 Center for Textile science and engineering, PNC Labs Inc., Osan 18104, Korea; hyeongyeol.choi@pnclabs.co.kr; 3 Department of Chemical Engineering, Hanyang University, Seoul 133-791, Korea; seongoh@hanyang.ac.kr; 4 Department of Chemistry and Chemical Engineering, Inha University, Incheon 22212, Korea; 5 Korea Institute of Industrial Technology, 143, Hanggaulro, Sangnok-gu, Ansan-si, Gyeonggi-do 15588, Korea

**Keywords:** electrospinning, nanofiber, inorganic nanoparticle adsorption, tri-layered structure, post-electrospinning treatment, dispersibility

## Abstract

We demonstrate a sequential electrospinning process involving the adsorption of ZnO nanoparticles on the surface of bio-based polyester, which is a terpolyester of a renewable isosorbide (ISB) monomer, ethylene glycol, 1,4-cyclohexane dimethanol, and terephthalic acid, the-so-called PEICT, to fabricate stable ZnO nanoparticles/PEICT nanofiber composite system protected with other two PEICT nanofiber mats. We found that post-electrospinning treatment with a particular solvent was effective to remove a residual solvent molecule in the PEICT nanofibers, which induced significant aggregation of the nanoparticles, leading to non-uniform distribution of the particles on the surface. Sequential electrospinning of the PEICT solution to sandwich ZnO nanoparticle-decorated PEICT nanofiber mat enabled to attain protected the inorganic/organic hybrid nanofiber mat, improving the long-term stability, and the reproducibility of the inorganic particles decorated nanofiber fabrication.

## 1. Introduction

Nanofiber mat, exhibiting a large surface area, high porosity, small pore size, and other advantageous features, has been widely utilized for a variety of applications such as water filtration membranes, drug delivery, tissue engineering, and wound dressing [1,2,3,4]. Nanofiber has also attracted significant attentions in the fields of composite technology due to the facile process for loading various particles in or on nanofibers. However, there are challenges for uniform incorporation of particles on nanofibers because particles tend to inherently agglomerate on account of van der Waals force, surface electrostatic charge, and high surface energy [5]. The poor dispersibility of the particles in solvents that are used for nanofiber fabrication and in the polymer matrix of fabricated nanofiber typically leads to the formation of large clusters, a decrease in surface area, and inhomogeneous distribution of the particles, ultimately resulting in the degradation of the performance in desired applications [6,7]. Hence, an even distribution of particles in or on the nanofiber is critical for the applicability of nanofibers in composite technology.

Up to now, a number of methods for the incorporation of particles on nanofibers have been reported to fabricate well-defined composite nanofibers [8,9,10]. Specifically, for electrospinning to form nanofibers, two methods have been employed: (i) the use of a polymer solution where the particles are dispersed in, and (ii) the immersion of prepared nanofibers in the medium where the particles are dispersed [9]. The use of a blending solution for electrospinning is highly effective for incorporating the particles inside of nanofibers; however, the major portion of particles are not exposed to the environments and therefore, the applicability of the particles is highly limited. Moreover, the particles are readily aggregated during the nanofiber formation process, significantly affecting the morphology of the composite nanofibers that is directly related to its physical properties [11]. The immersion method is highly simple and effective for the particles to be adsorbed on the surface of the nanofibers [12,13]. However, the detachment of the particles from the surface of nanofibers readily occurs, and agglomeration of particles is also still often observed, which is not desirable.

**Figure 1:**
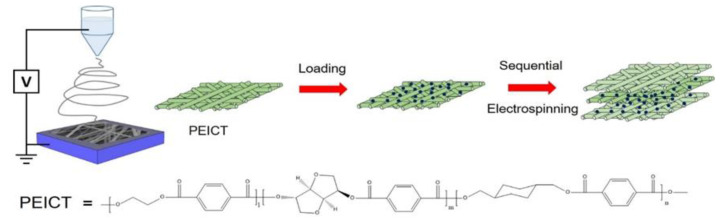
The fabrication process of a tri-layer structure, ZnO/PEICT composite nanofiber mat sandwiched with top and bottom PEICT nanofiber mats via the processes of post-electrospinning treatment, immersion for nanoparticle loading, and sequential electrospinning.

Herein, we demonstrate the tri-layered structure where an electrospun nanofiber mat of a ZnO nanoparticle-adsorbed bio-based polyester called PEICT (terpolyester of a renewable isosorbide (ISB) monomer, ethylene glycol, 1,4-cyclohexane dimethanol, and terephthalic acid) was sandwiched with two PEICT nanofiber mats via sequential electrospinning. According to our previous study, the incorporation of isosorbide does not only endows the polymer with environmental friendliness, but also provide the advantages of high glass transition temperature (T_g_), excellent mechanical strength, and transparency [14]. ZnO, an n-type semiconductor in the undoped form with a bandgap of 3.37 eV, was used as the nanoparticles to provide functionality on the polymeric nanofiber [15]. Due to its cost-effectiveness, nontoxicity, and excellent photocatalysis capability [16], it can be utilized as an effective additive for widening the application of polymers such as medical devices, self-cleaning textiles, breathable masks, and artificial implants [17,18,19,20,21,22,23,24]. We show that the instability and agglomeration issues of the adsorbed ZnO nanoparticles were addressed by simple post-electrospinning treatment with a solvent to remove residual solvent molecules in the as-spun PEICT nanofiber. With the treatment, we found that the particles were uniformly adsorbed without significant particle aggregation. Construction of a layer-by-layer structure with nanofibers through the sequential electrospinning process is a possible way to address the issue of the detachment of physically adsorbed particles on the nanofiber surface. By sandwiching the PEICT nanofiber mat with uniformly adsorbed ZnO nanoparticles with two nanofiber mats, the ZnO nanoparticles on nanofiber can be protected from the detachment issue and eventually, the platform of stable functional nanofiber composites is feasible (Figure 1).

## 2. Materials and Methods

### 2.1. Materials

Pellet-type PEICT (ECOZEN T95) (M_w_ = 65,400 g/mol) was kindly provided by SK Chemicals (Korea). Chloroform (99%) and trifluoroacetic acid (TFA, 98%) were purchased from Wako Chemicals (Japan). Zinc acetate dehydrate (Zn(CH_3_COO)_2_∙2H_2_O, Sigma-Aldrich, Milwaukee, WI, USA), potassium hydroxide (KOH, Junsei Chemical Co. Ltd., Tokyo, Japan), anhydrous ethanol (99.9%, Daejung Chemicals & Metals Co. Ltd., Siheung, Korea), methanol (CH_3_OH, OCI Co. Ltd., Seoul, Korea), and *n*-butanol (Sigma-Aldrich, Milwaukee, WI, USA) were used to synthesize the ZnO nanoparticles. The reagents were used without further purification, or as otherwise noted.

### 2.2. Characterizations

The morphology of the fabricated nanofibers was examined using scanning electron microscopy (SEM, JSM-6010LA, JEOL Ltd., Tokyo, Japan) and transmission electron microscopy (TEM, JEM-2100, JEOL Ltd., Japan). X-ray diffraction (XRD, Miniflex 300, Rigaku Co. Ltd., Tokyo, Japan) analysis was performed using CuKα radiation operated at 30 kV and 500 mA to examine the crystallinity of samples in the 2θ range of 10° to 80°.

### 2.3. ZnO Nanoparticle Fabrication

ZnO nanoparticles were synthesized by a sol-gel process in a methanol medium, utilizing zinc acetate dehydrate (Zn(CH_3_COO)_2_∙2H_2_O) and potassium hydroxide [25]. Ten mmol of zinc acetate dehydrate was added in 100 mL of ethanol and the mixture was stirred at 60 °C for 30 min, resulting in a transparent solution. The solution of potassium hydroxide (20 mmol) in ethanol (50 mL) prepared by stirring at 60 °C for 30 min was added dropwise to the transparent zinc acetate dehydrate/ethanol solution. Upon completion of the addition, the reaction mixture was stirred at 60 °C for 3 h, followed by additional stirring at room temperature for 4 h. ZnO nanoparticles were isolated using a centrifuge at 3000 rpm for 15 min, and the solvent was exchanged with *n*-butanol. The final concentration of the nanoparticle dispersion was adjusted to 6.57 wt.% of the ZnO nanoparticles (the size less than 10 nm) in *n*-butanol.

### 2.4. Nanofiber Fabrication

In order to homogeneously dissolve PEICT, a binary solvent system was employed by mixing TFA and chloroform with the volume ratio of 7/3. A certain amount of PEICT pellet was then added to the mixed solvent system to achieve a 10 wt.% polymer solution. The mixture was stirred vigorously for 24 h until all solids disappeared and the solution became clear. The electrospinning apparatus was built with a pump (KDS-100, KD Scientific, Holliston, MA, USA) to control the solution flow rate, a collector, and a syringe containing PEICT solution. The needle with an inner diameter of 0.8 mm was attached to the syringe. Afterward, the syringe with the needle was mounted as a spinneret on the pump. The processing parameters for the electrospinning were adjusted to the voltage of 15 kV, the distance from tip to collector of 20 cm, temperature of 25 °C, and relative humidity of approximately 50%. The electrospinning was continuously performed for 12 h to fabricate the PEICT nanofiber web (PEICT NF) with high thickness uniformity.

PEICT NF/ZnO composite samples were prepared by the simple immersion approach with or without post-electrospinning treatment of the as-spun PEICT nanofiber mat with ethanol (simple immersion of as-spun PEICT nanofiber mat in ethanol for 5 min.). In particular, the PEICT nanofiber sheet with the dimensions of 20 cm × 20 cm (length × width) was immersed into the tray containing 200 mL of the ZnO nanoparticle solution. The tray containing the solution and the PEICT nanofiber mat was gently agitated at 150 rpm for 24 h by a shaking apparatus (Rotary shaker NA-301N, Nissin, Japan) at room temperature. The resulting PEICT/ZnO composite nanofiber sample was then dried at room temperature for 24 h.

To encompass the PEICT NF/ZnO composite with PEICT nanofiber mats, the same electrospinning process was additionally performed to cover the front side and the rear side of the PEICT NF/ZnO composite sample. The fabricated PEICT/ZnO composite was fixed on the collector, then the electrospinning of the PEICT solution was conducted for 12 h on the front side, and for an additional 12 h on the other side to secure the structure of the sandwiched ZnO-containing PEICT nanofiber mat layer by two other PEICT nanofiber mat layers with a similar thickness. The tri-layered nanofiber mat was washed thoroughly with ethanol and water, and dried at room temperature.

## 3. Results

### 3.1. The Effect of Post-Electrospinning Treatment of Nanofibers on Composite Structure Formation

**Figure 2:**
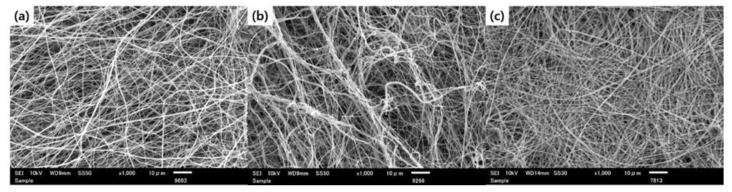
SEM images showing morphologies of (**a**) as-spun PEICT nanofiber, (**b**) PEICT/ZnO nanofibers without post-electrospinning treatment with ethanol, and (**c**) PEICT/ZnO composite nanofibers by post-electrospinning treatment with ethanol and subsequent loading of ZnO nanoparticles.

In the electrospinning process, an electrically charged polymer solution droplet on a spinneret is stretched out to form nanofibers. The stretched fibrous objects travel through air, and are eventually collected on a collector surface. During the flight, the solvent molecules in the nanofiber evaporate, leading to the formation of polymeric fibers. However, trace amounts of solvent molecules are retained in the nanofiber as complete evaporation of all of the solvent molecules cannot occur and some molecules are trapped in the nanofiber. Therefore, remaining solvent molecules possibly affect the morphology and state of resulting nanofibers and related composite preparation processes such as the immersion process. To examine this aspect, morphological studies of nanofibers were conducted using SEM, particularly to in investigate the effect of post-electrospinning treatment with ethanol before the nanoparticle loading process. As-spun PEICT nanofibers show a typical nanofibrous structure with bead-free, smooth, uniform, and randomly oriented features. However, the nanofibers after immersion in the ZnO solution without pre-washing exhibited significant agglomeration of nanofibers and as a consequence, the nanofiber mat was shrunk. (Figure 2b) In contrast, the post-electrospinning treatment with ethanol before ZnO loading did not lead to nanofiber agglomeration, as shown in Figure 2c.

**Figure 3:**
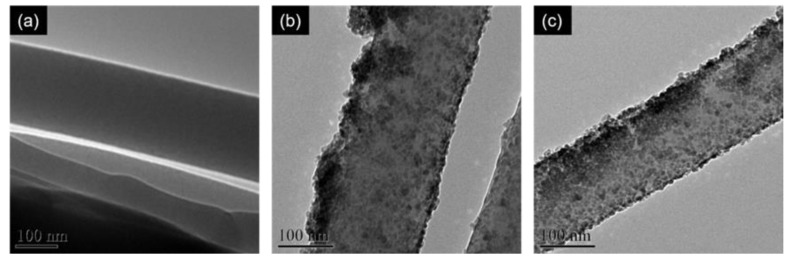
TEM images showing the surface morphologies of (**a**) an as-spun PEICT nanofiber, (**b**) a PEICT/ZnO composite nanofiber without post-electrospinning treatment with ethanol, and (**c**) PEICT/ZnO composite nanofibers treated with ethanol before ZnO nanoparticle loading.

Furthermore, the distribution of nanoparticles on the nanofiber was also largely affected by the post-electrospinning treatment. As shown in Figure 3b,c, both PEICT/ZnO composite nanofibers prepared with and without post-electrospinning ethanol treatment exhibited the presence of adsorbed ZnO nanoparticles with the diameter of 5–10 nm on the surface of the nanofibers; however, the state of particle aggregation was observed to be well-distinguished. It is clear that untreated PEICT nanofibers likely induce the aggregation of ZnO nanoparticles with a poor dispersibility. In contrast, the post-electrospinning treatment of PEICT nanofibers results in the effective adsorption of ZnO nanoparticles without severe aggregation, and with relatively uniform distribution over the surface.

This phenomenon is likely attributed to the residual TFA molecules upon the electrospinning process. It is generally known that metal ions or particles are aggregated in acidic conditions [26]. When being treated with a ZnO solution in ethanol without complete removal of the residual solvent, the remaining TFA molecules inside the nanofibers diffuse instantly to the medium and interact with ZnO nanoparticles on the surface. Leached TFA molecules from the nanofibers can also induce a significant aggregation of the ZnO nanoparticles, which was clearly observed in Figure 3b. In addition, nanoparticles on a PEICT nanofiber can interact with nanoparticles on another PEICT nanofiber, resulting in the agglomeration of nanofibers, which was seen in Figure 2b. Therefore, the results strongly suggest that the post-electrospinning solvent treatment process plays an essential role in attaining well-defined composite nanofiber mats with a homogeneous distribution of nanoparticles on the nanofiber surface.

### 3.2. Envelopment of ZnO Nanoparticles on Nanofibers by the Formation of Layer-by-Layer Structure

**Figure 4:**
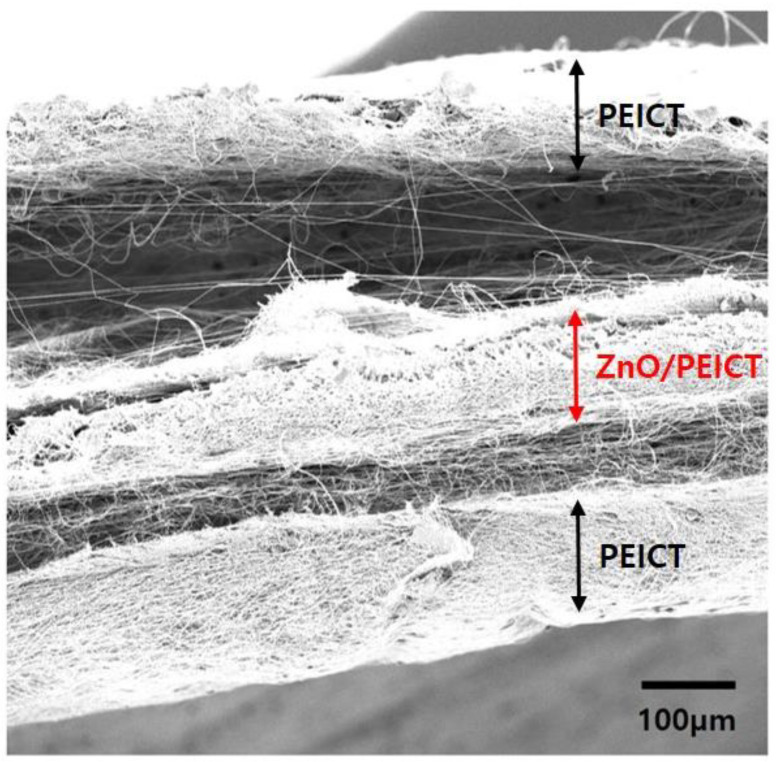
SEM image showing cross-sectional view of the tri-layered nanofiber mat structure prepared by sequential electrospinning.

The method for incorporating nanoparticles in nanofibers has been widely investigated and utilized in various applications (e.g., biomedical and energy storage fields [27,28,29]). One of the most simple, but effective approaches for loading nanoparticles is the immersion of the nanofiber mat into a nanoparticle solution, as described above. However, since it relies on the physical interaction of the particles with the nanofiber surface, the detachment of the nanoparticles from the nanofibers is often observed. Therefore, it requires some protection to prevent the nanoparticle desorption. The sequential electrospinning method is one of the candidates to hold nanoparticles in the nanofiber mat. Using this approach, a sandwich structure can be attained by further electrospinning processes on the nanofiber composite mat (i.e., the top and the bottom PEICT nanofiber mat layers, and the middle layer of a ZnO loaded PEICT nanofiber composite mat). These top and bottom layers effectively hinder nanoparticles to wander from the middle layer by external influences. The cross-sectional SEM image in Figure 4 shows the morphology of a tri-layered nanofiber mat with the middle layer of a ZnO/PEICT nanofiber composite mat. The sequential electrospinning processes were successful in preparing the tri-layered structure easily on the previously fabricated nanofiber mat. It was notable that immediate electrospinning of the PEICT nanofiber helps the layers to interact between layers.

**Figure 5:**
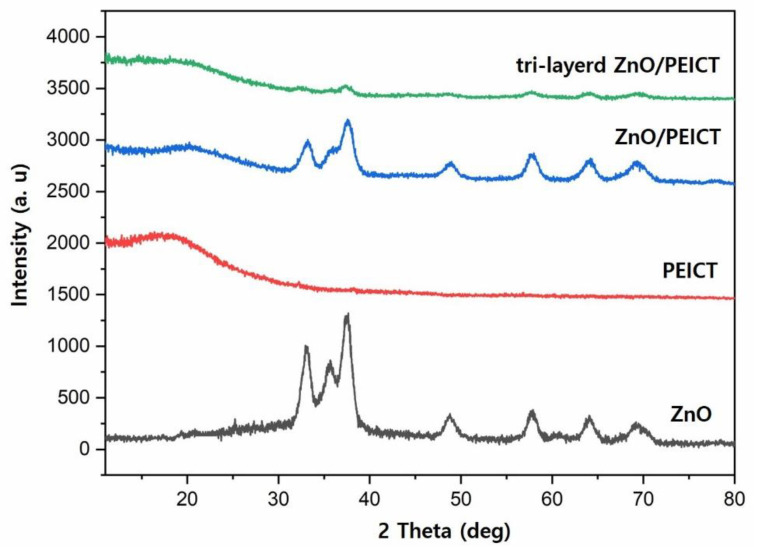
XRD patterns of the ZnO nanoparticle, the PEICT nanofiber mat, the ZnO/PEICT composite nanofiber upon the removal of TFA, and tri-layered nanofiber mat by sequential electrospinning process.

Nanoparticle encapsulation between nanofiber matrices was further confirmed by XRD analysis. Figure 5 shows the wide-angle X-ray diffraction spectra of the as-spun PEICT nanofiber, as-synthesized by the ZnO nanoparticle, the ZnO/PEICT composite nanofiber mat prepared with post-electrospinning solvent treatment, and the tri-layered nanofiber mat fabricated by sequential electrospinning process. The as-spun PECIT nanofiber mat showed a typical amorphous feature due to the ISB unit, which has a V-shaped structure with a 120° angle between the rings. The uniqueness of the ISB unit makes it difficult to induce the crystallization of the polymer chain [14,30]. Therefore, it only showed a slight shoulder peak at approximately 18°, indicating that the PEICT nanofiber is amorphous. On the other hand, the XRD pattern of the ZnO nanoparticle sample exhibited clear crystalline peaks at 2θ values of 31.76°, 34.51°, 36.33°, 47.69°, 56.86°, 63.10°, 66.68°, 68.22°, and 69.32°, which correspond to the (100), (002), (101), (102), (110), (103), (200), (112), and (201) planes of the hexagonal wurtzite structure of ZnO [31]. It was found that there was no significant shift in the peak position of ZnO upon the adsorption on the PEICT nanofiber surface, indicating that the ZnO nanoparticles were not only well-distributed in the PEICT nanofiber mat, but the crystal structure of the ZnO nanoparticle was also preserved during nanoparticle loading by the immersion process with post-electrospinning solvent treatment. More importantly, the XRD pattern of ZnO nanoparticles was also observed in the tri-layered ZnO/PEICT composite nanofiber mat, matching the peaks observed in the ZnO nanoparticle sample, even if possibly harmful processes including the use of different solvents are involved. Generally, adsorbates incorporated by the immersion process are readily detached under the washing process since they are physically stuck on the polymeric matrix. However, the sequential electrospinning technique prevents the detachment of nanoparticle adsorbates by hindering separation from the top and bottom nanofiber matrices. In addition, the top and bottom PEICT nanofiber mats allow us to preserve its remarkable properties, which can make different applications feasible [14]. Therefore, the current results highlight that a well-defined nanoparticle-incorporated nanofiber mat can be prepared with this easy, intuitive, and effective process for loading and sustaining additives on polymeric nanofibers.

## 4. Conclusions

We showed the fabrication of the ZnO incorporated PEICT nanofiber and investigated the effect of the removal of residual solvent before the adsorption of ZnO nanoparticles by immersion. The residual TFA molecule in or on the PEICT nanofiber made ZnO nanoparticles aggregated on the surface, leading to uneven distribution on the nanofiber surface. By the post-electrospinning solvent treatment process, residual TFA was effectively removed and therefore, the uniform distribution of ZnO nanoparticles without aggregation on the surface of the PEICT nanofiber was feasible. These results offer an effective particle loading approach with an immersion process of electrospun nanofibers. Furthermore, we highlighted the benefit of a sequential electrospinning method for protecting the nanoparticles incorporated by the immersion process. The sequential electrospinning provides the sandwich structure consisting of PEICT nanofiber mats, where the ZnO nanoparticle-decorated PEICT mat lies between two PECIT nanofiber mats. This sandwich structure can prevent the detachment of ZnO nanoparticles by capturing nanoparticles in the space made between two nanofiber layers. The current results further highlight that the combination of a post-electrospinning solvent treatment process, immersion for particle loading, and sequential electrospinning can be a useful strategy for controlling the complexity of the nanofiber composite material, particularly in expanding the applicability of ZnO/PEICT nanofiber as an organic/inorganic hybrid composite material.
